# Supplementary material for: Cation-driven phase transition and anion-enhanced kinetics for high energy efficiency zinc-interhalide complex batteries
Source: Nat Commun. 2025 May 17;16:4586. doi: 10.1038/s41467-025-59894-w (PMC12085677; doi:10.1038/s41467-025-59894-w)
Supplement: Supplementary file 1 — Supplementary Information [file 41467_2025_59894_MOESM1_ESM.pdf]

# Supplementary Information

## Cation-Driven Phase Transition and Anion-Enhanced Kinetics for High Energy Efficiency Zinc-Interhalide Complex Batteries

*Wei Zhong*[1,2,3], *Hao Cheng*[1,2,3,*], *Shichao Zhang*[1], *Laxi Li*[1,2,3], *Chaoqiang Tan*[1,2], *Wei Chen*[4] and *Yingying Lu*[1,2,3,*]

1. State Key Laboratory of Chemical Engineering, Institute of Pharmaceutical Engineering, College of Chemical and Biological Engineering, Zhejiang University, Hangzhou 310027, China.
2. ZJU-Hangzhou Global Scientific and Technological Innovation Center, Zhejiang University, Hangzhou 311215, China.
3. Institute of Wenzhou, Zhejiang University, Wenzhou 325006, China.
4. Department of Applied Chemistry, School of Chemistry and Materials Science, Hefei National Research Center for Physical Sciences at the Microscale, University of Science and Technology of China, Hefei, Anhui 230026, China.

[*]Corresponding author. *E-mail:* Bob_hao@zju.edu.cn (Hao Cheng); yingyinglu@zju.edu.cn (Yingying Lu)

**This PDF file includes:**

Supplementary Figs. 1 to 55.
Supplementary Notes 1 to 3.
Supplementary Tables 1 to 6.

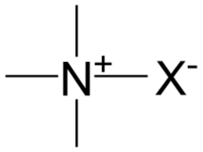


**Supplementary Fig. 1.** Chemical structure of TMAX.

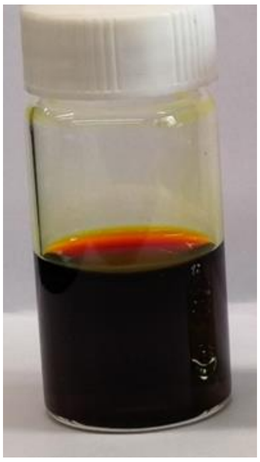

**Supplementary Fig. 2.** Photograph of $KI_3$ solution.

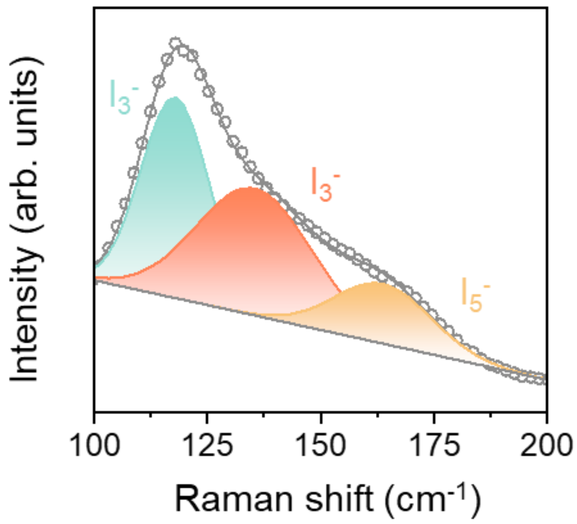


**Supplementary Fig. 3.** Fitted Raman spectra of $KI_3$ solution.

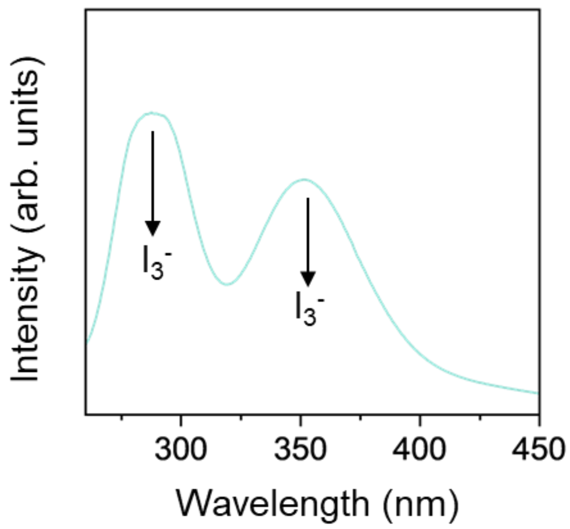


**Supplementary Fig. 4.** UV-vis spectra of $KI_3$ solution.

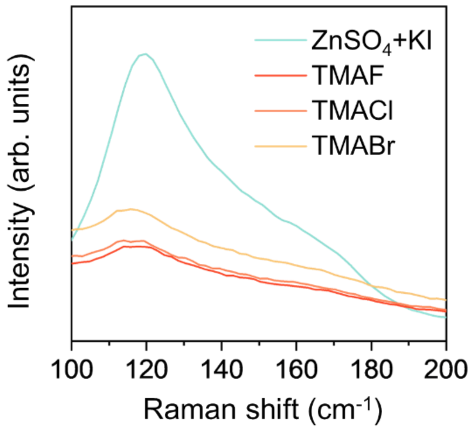


**Supplementary Fig. 5.** Raman spectra of the supernatant after mixing.

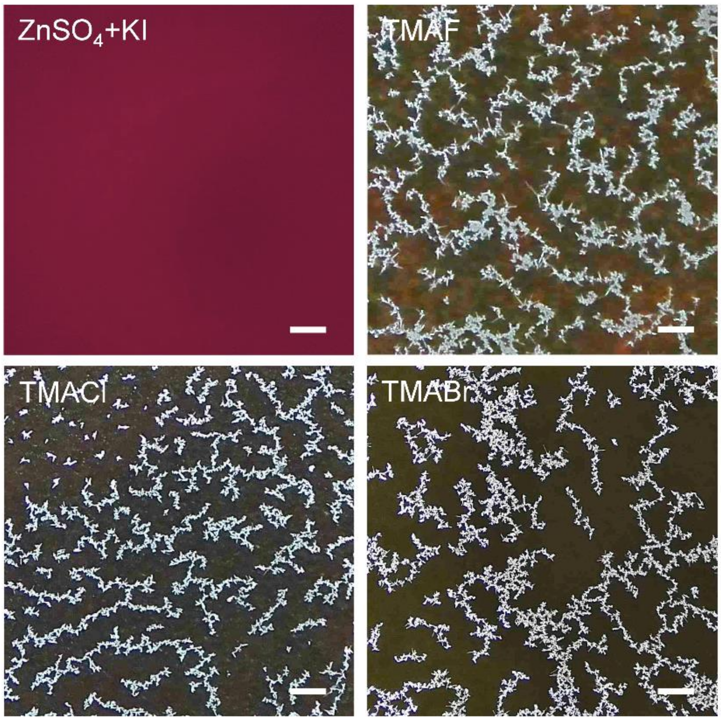


**Supplementary Fig. 6.** Visualization of association processes after mixing $ZnSO_4$+KI and TMAX into $I_3^-$ solutions for 15 seconds (scale bar= 0.5 mm).

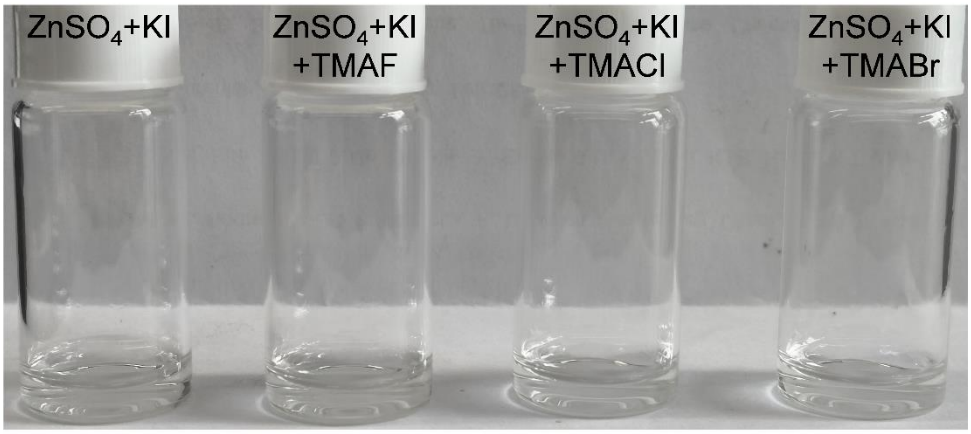


**Supplementary Fig. 7.** Photographs of the mixture of posolyte and negolyte.

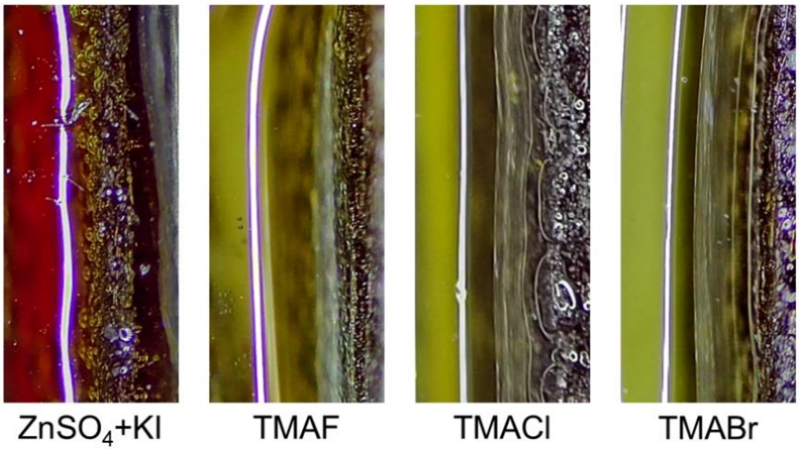


**Supplementary Fig. 8.** Optical images of the positive electrode-electrolyte interface after charging using different electrolytes.

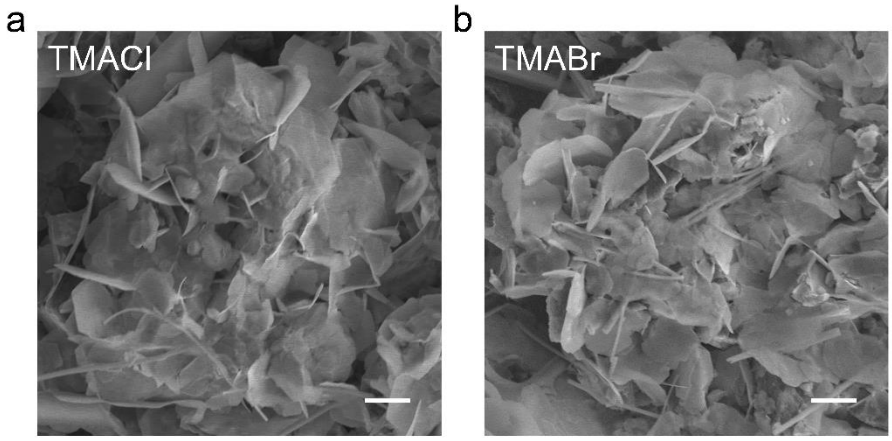


**Supplementary Fig. 9.** SEM images of positive electrodes charged to 1.6 V at a specific current of 0.2 A $g^{-1}$ using **a** TMACl and **b** TMABr electrolytes. Scale bar=1 μm.

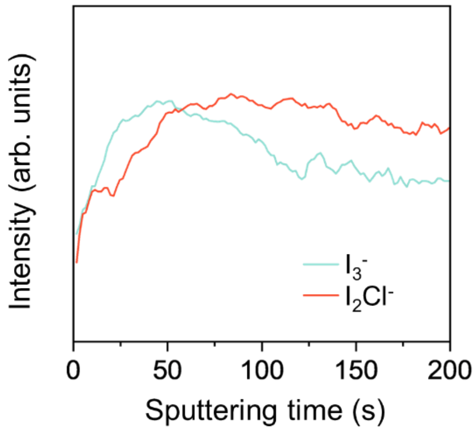


**Supplementary Fig. 10.** ToF-SIMS negative in-depth ion profiles for positive electrodes charged to 1.6 V at a specific current of 0.2 A $g^{-1}$ performed in TMACl electrolytes.

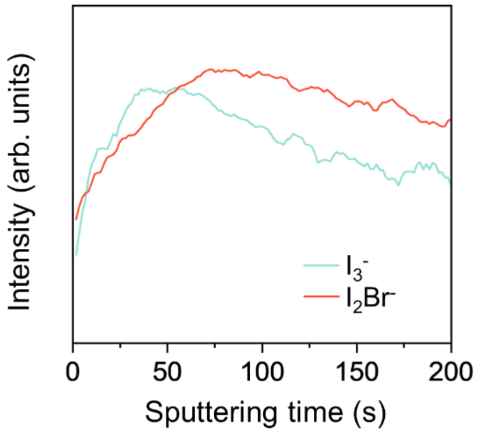


**Supplementary Fig. 11.** ToF-SIMS negative in-depth ion profiles for positive electrodes charged to 1.6 V at a specific current of 0.2 A $g^{-1}$ performed in TMABr electrolytes.

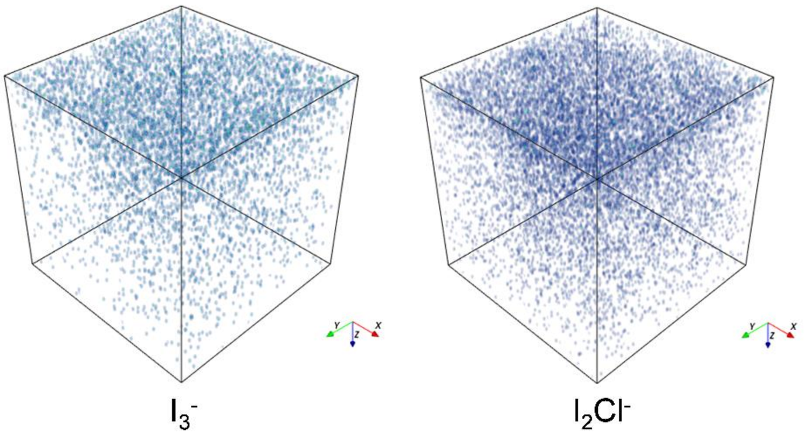


**Supplementary Fig. 12.** 3D ToF-SIMS visual maps for positive electrodes charged to 1.6 V at a specific current of 0.2 A $g^{-1}$ performed in TMACl electrolytes.

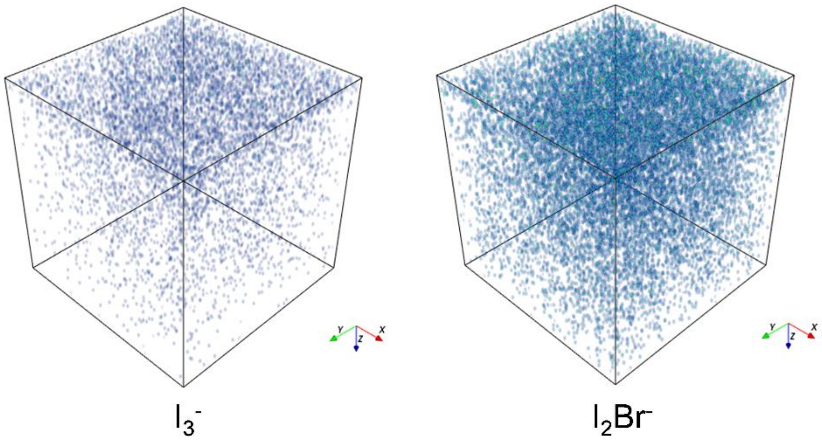


**Supplementary Fig. 13.** 3D ToF-SIMS visual maps for positive electrodes charged to 1.6 V at a specific current of 0.2 A $g^{-1}$ performed in TMABr electrolytes.

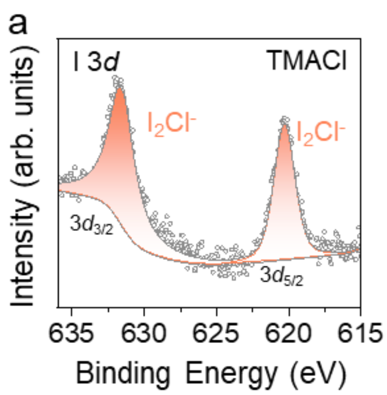


**Supplementary Fig. 14.** Fitted **a** I 3*d* and **b** Cl 2*p* XPS spectra of positive electrodes charged to 1.6 V at a specific current of 0.2 A $g^{-1}$ using TMACl electrolytes.

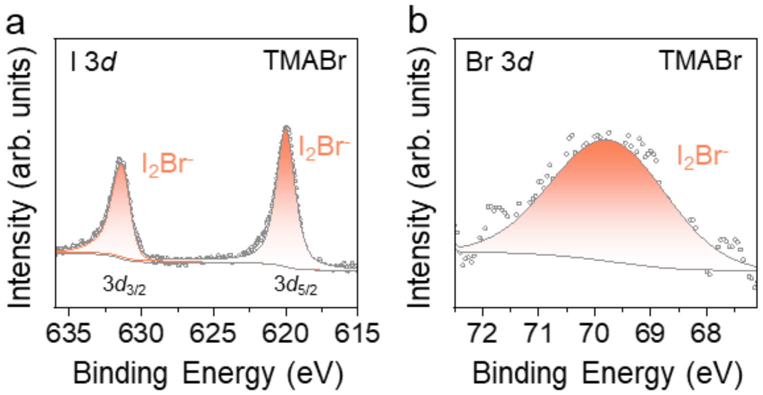


**Supplementary Fig. 15.** Fitted **a** I 3d and **b** Br 3*d* XPS spectra of positive electrodes charged to 1.6 V at a specific current of 0.2 A $g^{-1}$ using TMABr electrolytes.

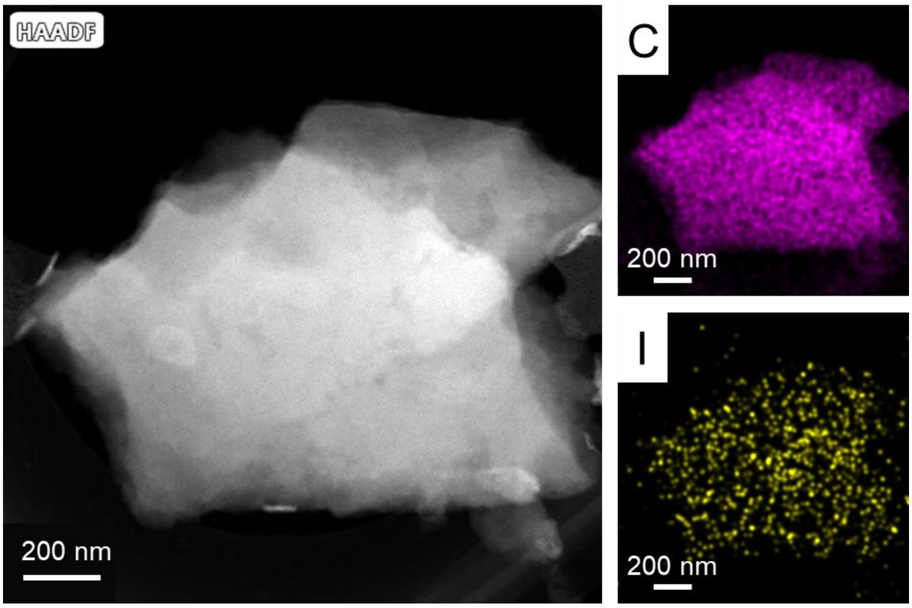


**Supplementary Fig. 16.** HRTEM images and EDS element mapping of positive electrodes charged to 1.6 V at a specific current of 0.2 A $g^{-1}$ using $ZnSO_4$+KI electrolytes.

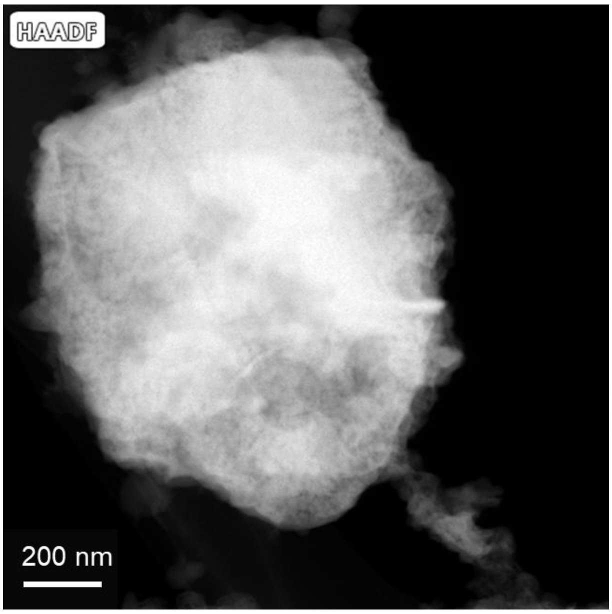


**Supplementary Fig. 17.** HRTEM images of positive electrodes charged to 1.6 V at a specific current of 0.2 A $g^{-1}$ using TMAF electrolytes.

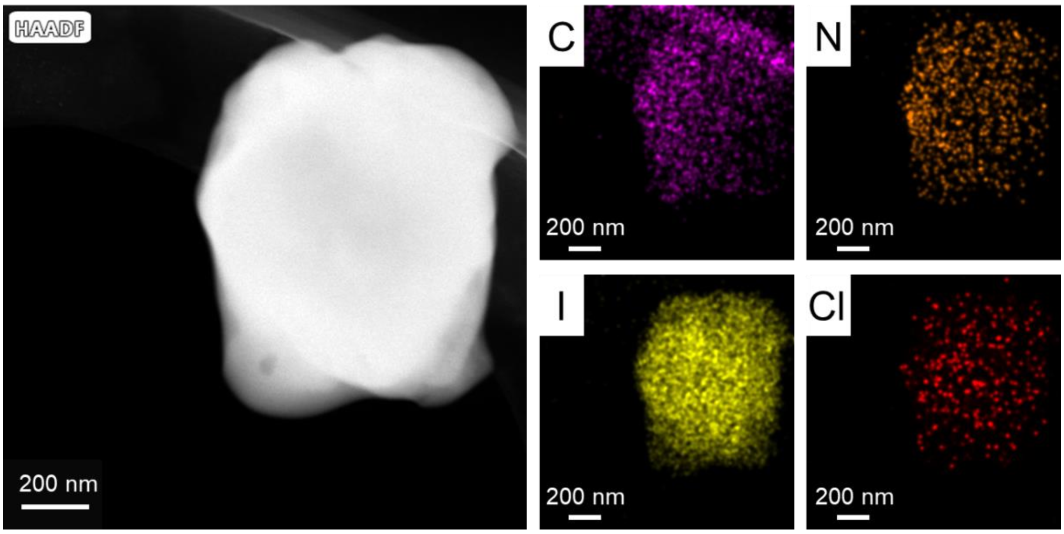


**Supplementary Fig. 18.** HRTEM images and EDS element mapping of positive electrodes charged to 1.6 V at a specific current of 0.2 A $g^{-1}$ using TMACl electrolytes.

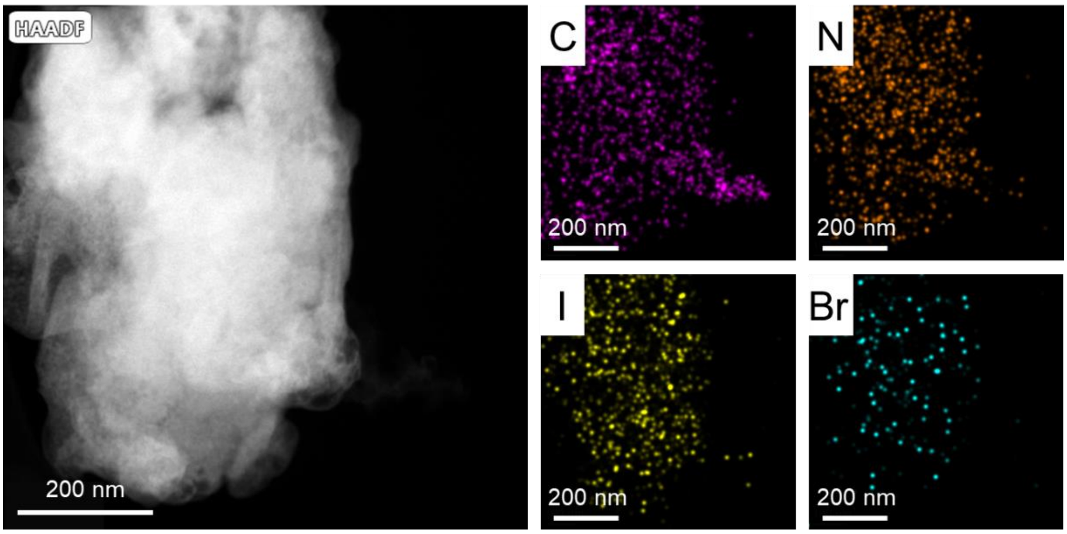


**Supplementary Fig. 19.** HRTEM images and EDS element mapping of positive electrodes charged to 1.6 V at a specific current of 0.2 A $g^{-1}$ using TMABr electrolytes.

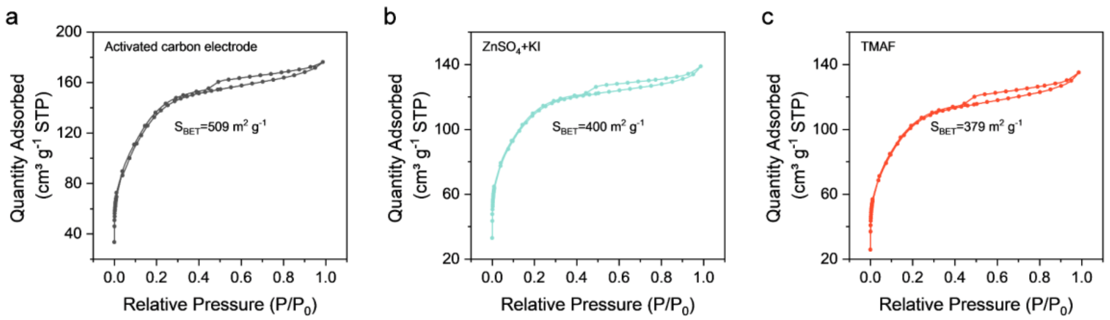


**Supplementary Fig. 20.** $N_2$ porosimetry measurements for **a** uncharged activated carbon electrodes and activated carbon electrodes charged to 1.6 V at a specific current of 0.2 A $g^{-1}$ in **b** $ZnSO_4$+KI and **c** TMAF electrolytes, respectively.

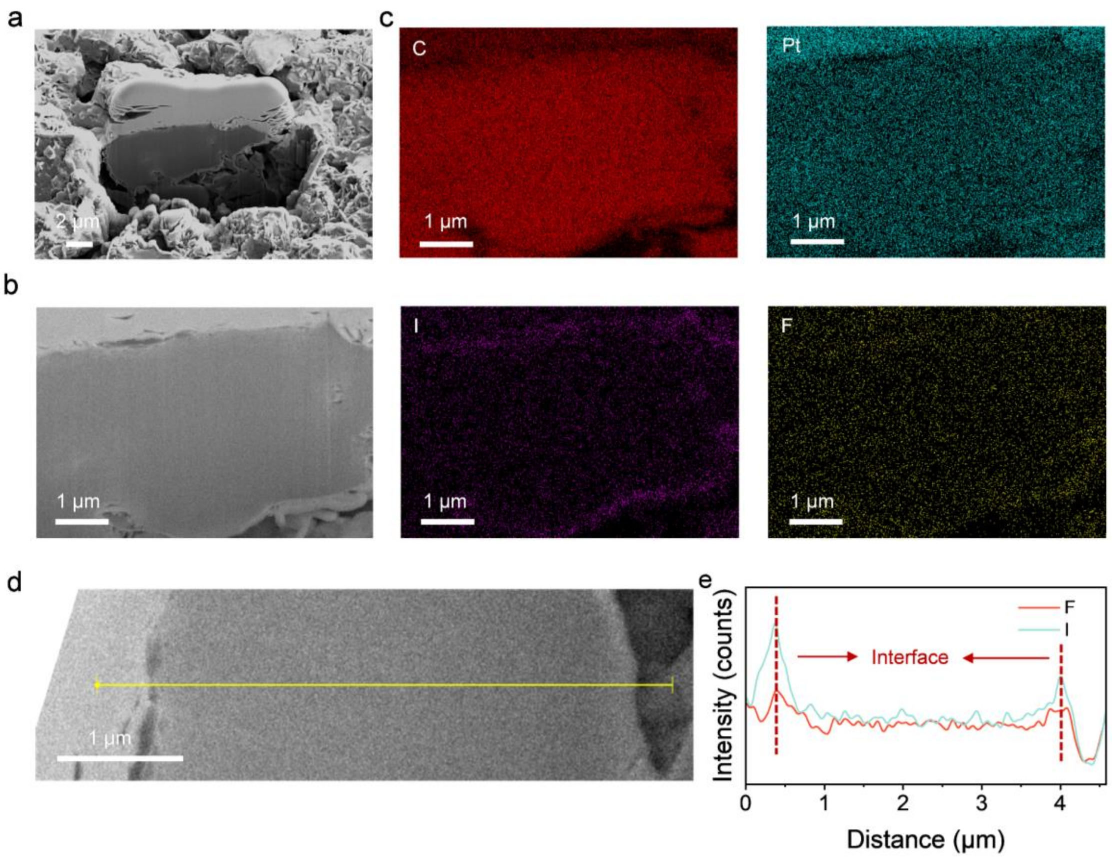


**Supplementary Fig. 21. a** Cross-section images of activated carbon after charging to 1.6 V at a specific current of 0.2 A $g^{-1}$ in TMAF electrolyte. **b** Magnified images of **a**. **c** EDS element mapping. **d, e** EDS line scanning.

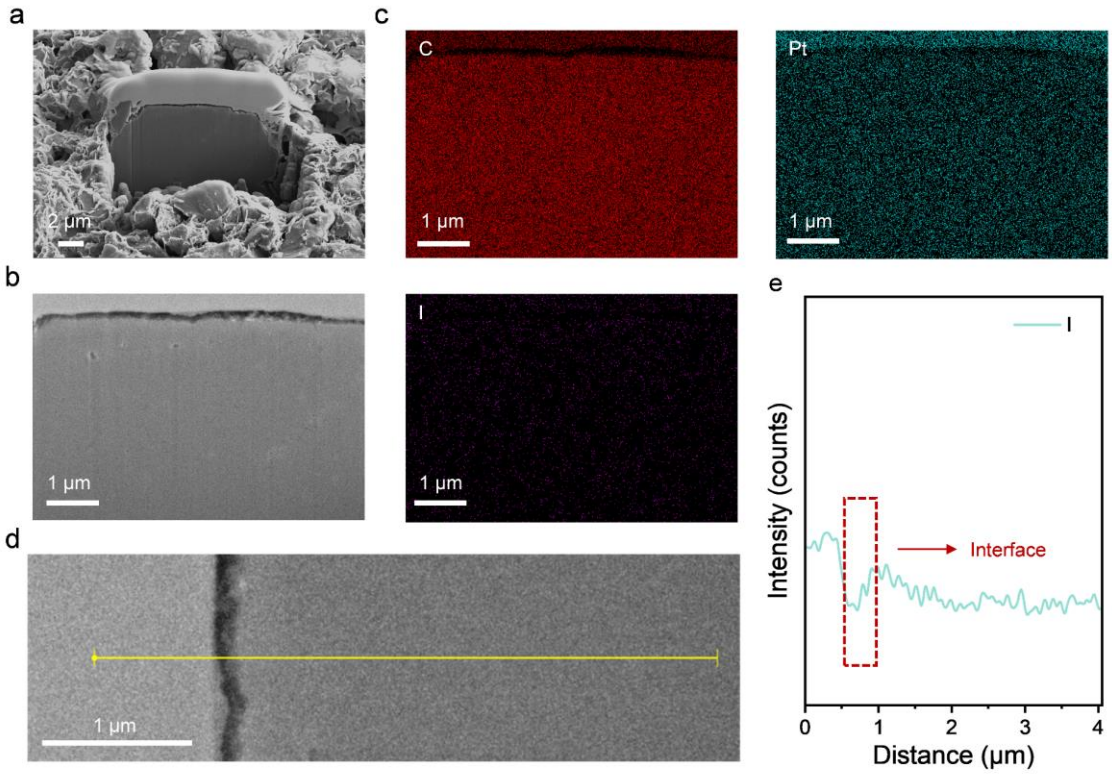


**Supplementary Fig. 22.** **a** Cross-section images of activated carbon after charging to 1.6 V at a specific current of 0.2 A $g^{-1}$ in $ZnSO_4$+KI electrolyte. **b** Magnified images of **a**. **c** EDS element mapping. **d, e** EDS line scanning.

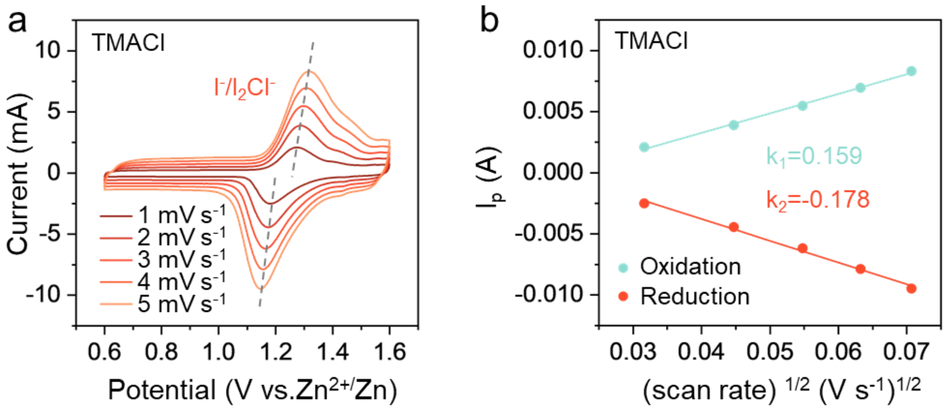


**Supplementary Fig. 23. a** CV profiles of ZICBs using TMACl electrolytes. **b** Relationship between peak current and square root of scan rate.

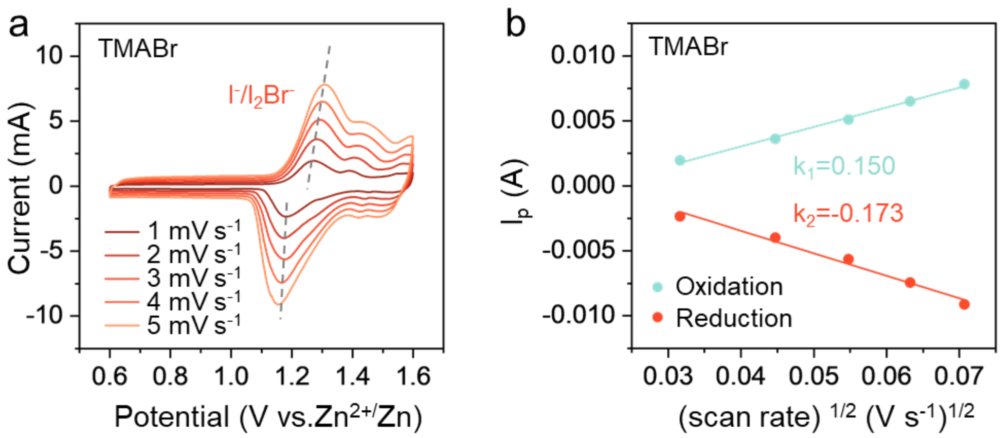


**Supplementary Fig. 24. a** CV profiles of ZICBs using TMABr electrolytes. **b** Relationship between peak current and square root of scan rate.

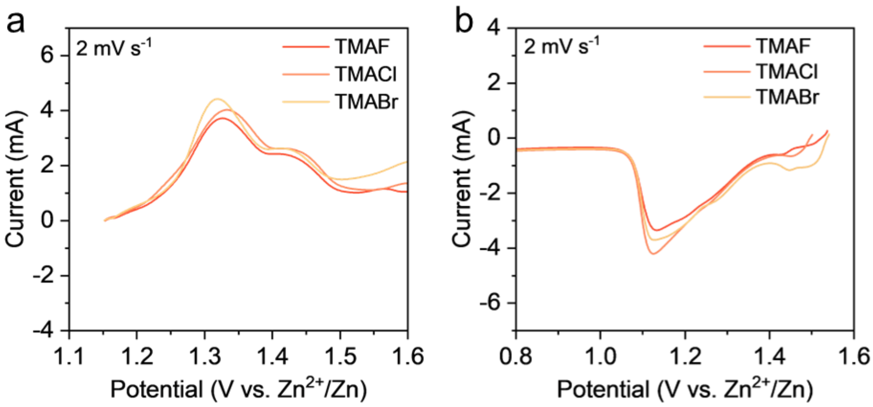


**Supplementary Fig. 25.** LSV curves of ZICBs toward **a** IOR and **b** IRR using TMAX electrolytes.

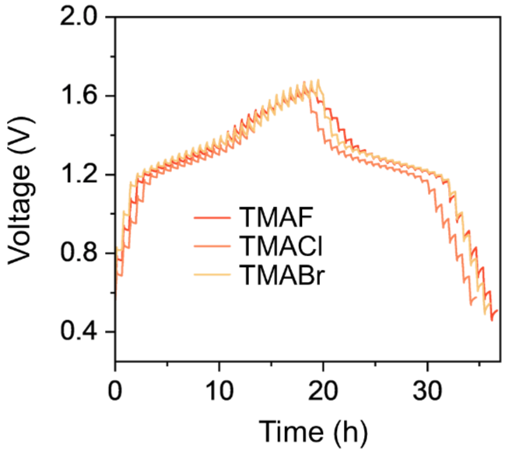


**Supplementary Fig. 26.** GITT curves of ZICBs using different electrolytes.

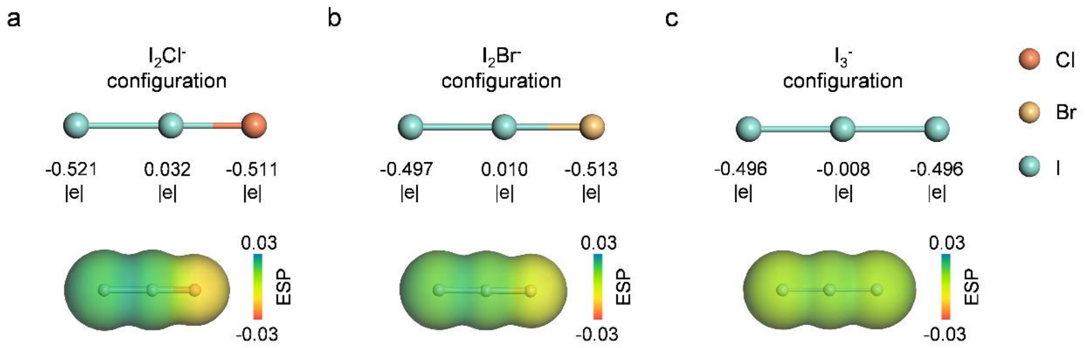


**Supplementary Fig. 27.** Configurational structures, valence states (atomic Mullikan charge) and ESP of **a** $I_2Cl^-$, **b** $I_2Br^-$ and **c** $I_3^-$.

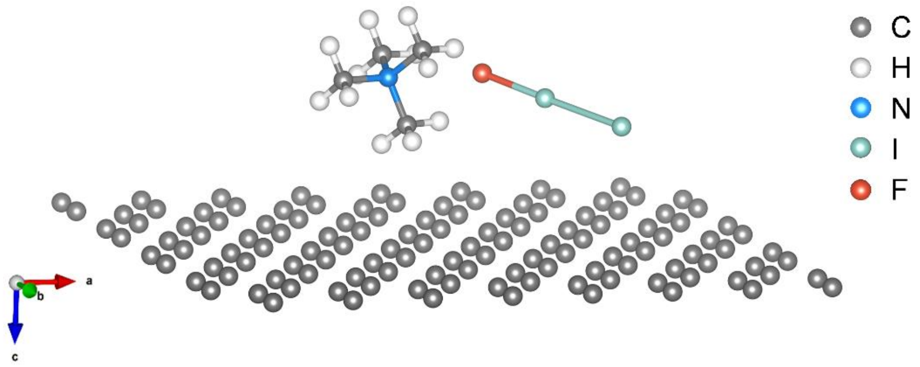


**Supplementary Fig. 28.** Adsorption of $TMAI_2F$ on carbon layer.

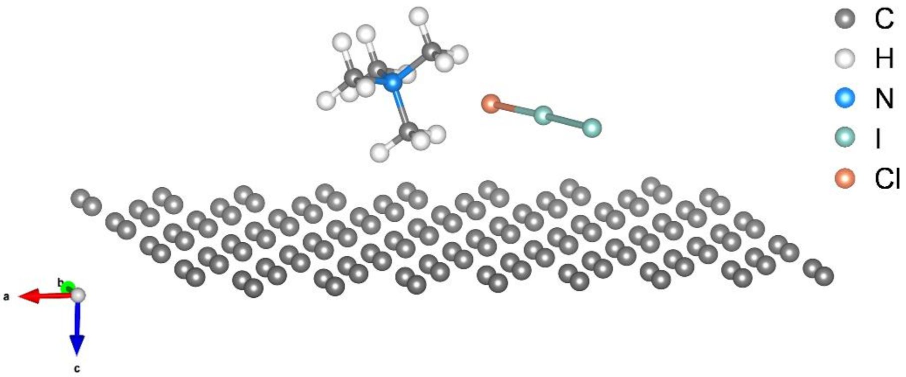


**Supplementary Fig. 29.** Adsorption of $TMAI_2Cl$ on carbon layer.

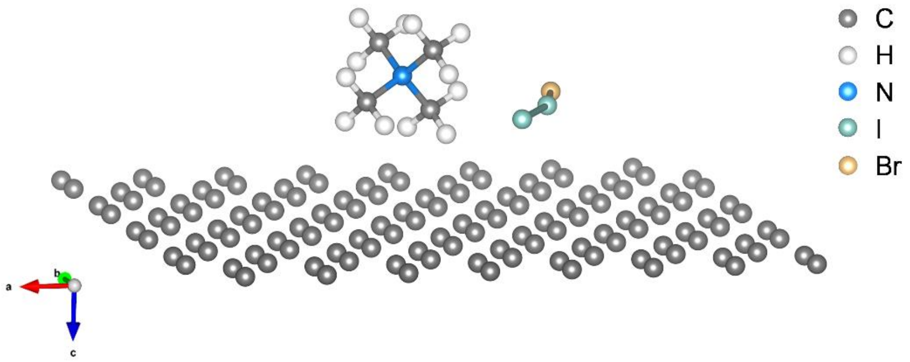


**Supplementary Fig. 30.** Adsorption of $TMAI_2Br$ on carbon layer.

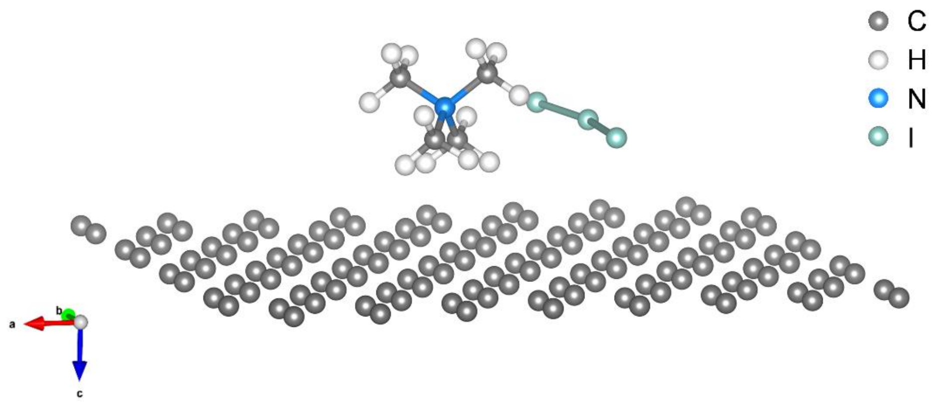


**Supplementary Fig. 31.** Adsorption of $TMAI_3$ on carbon layer.

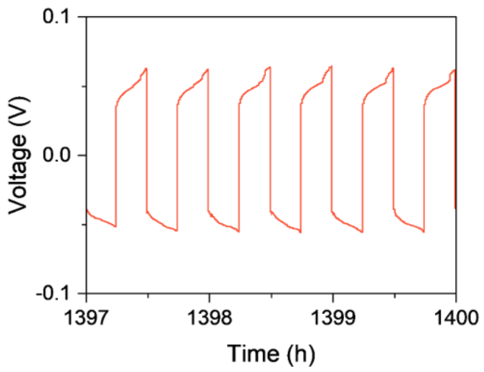


**Supplementary Fig. 32.** Enlarged views of galvanostatic Zn plating/stripping stability of symmetric Zn||Zn cells using TMAF electrolytes under 4 mA cm$^{-2}$, 1 mAh cm$^{-2}$ during 2794-2800 cycles.

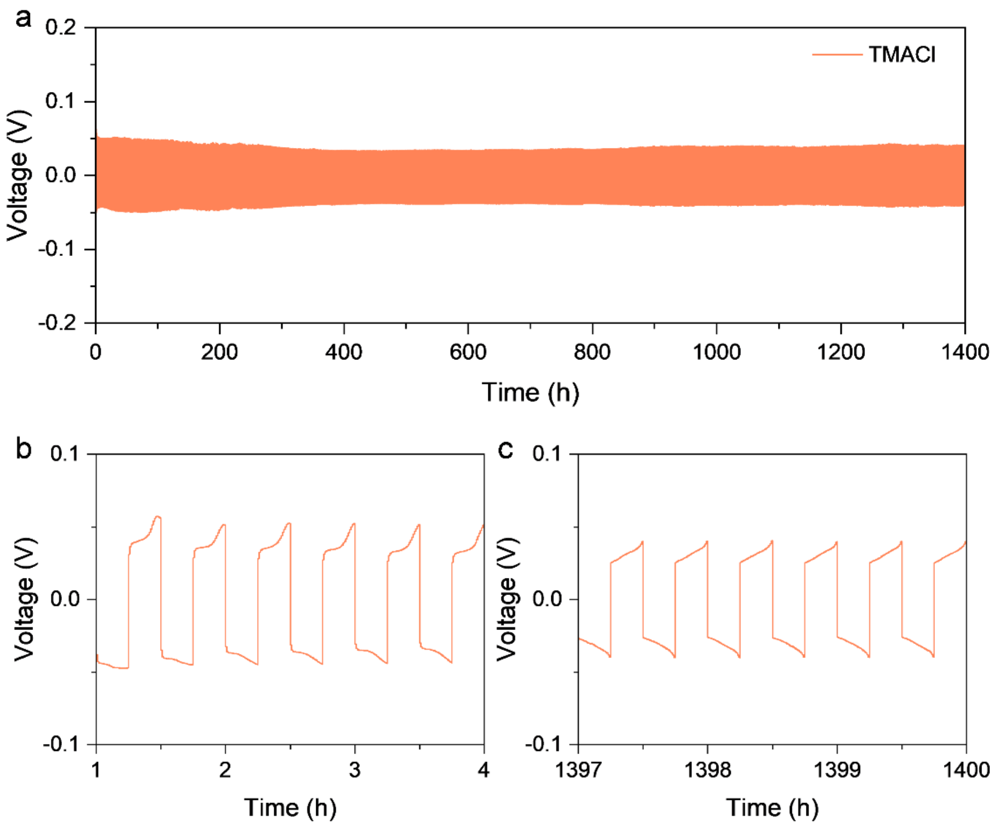


**Supplementary Fig. 33. a** Galvanostatic Zn plating/stripping stability of symmetric Zn||Zn cells using TMACl electrolytes under 4 mA $cm^{-2}$, 1 mAh $cm^{-2}$, tested at 30°C. Enlarged views of **b** 2-8 cycles and **c** 2794-2800 cycles.

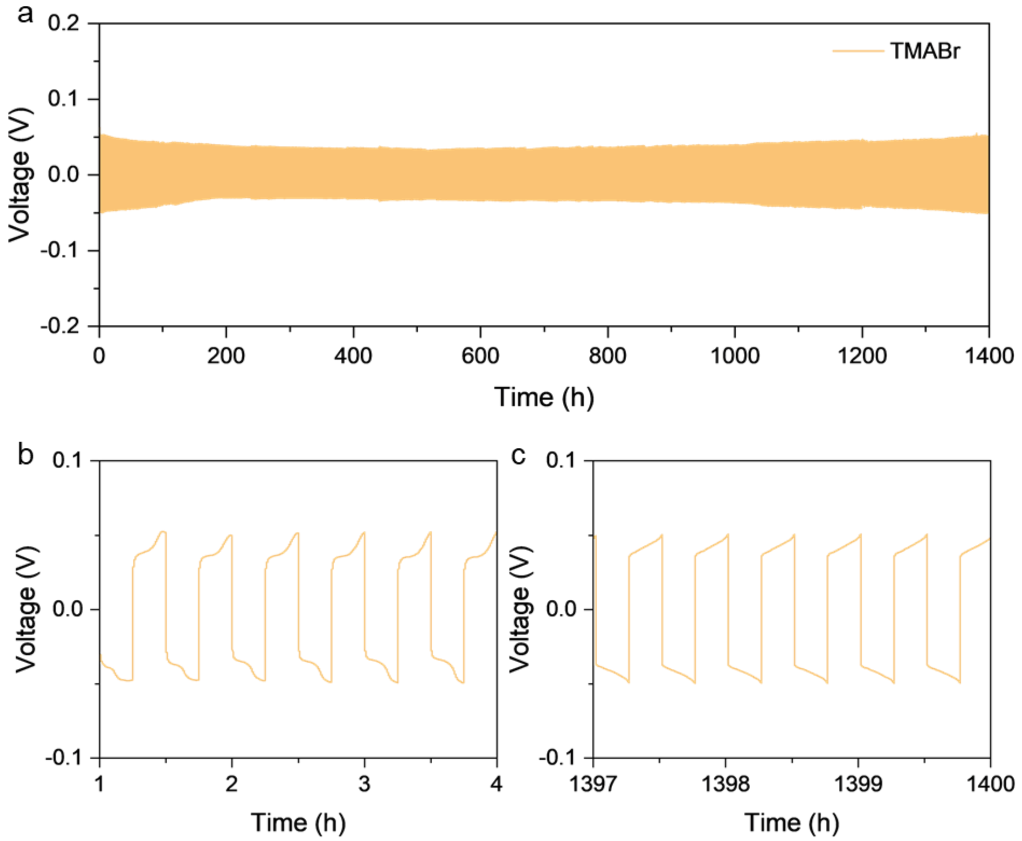


**Supplementary Fig. 34. a** Galvanostatic Zn plating/stripping stability of symmetric Zn||Zn cells using TMABr electrolytes under 4 mA cm$^{-2}$, 1 mAh cm$^{-2}$, tested at 30°C. Enlarged views of **b** 2-8 cycles and **c** 2794-2800 cycles.

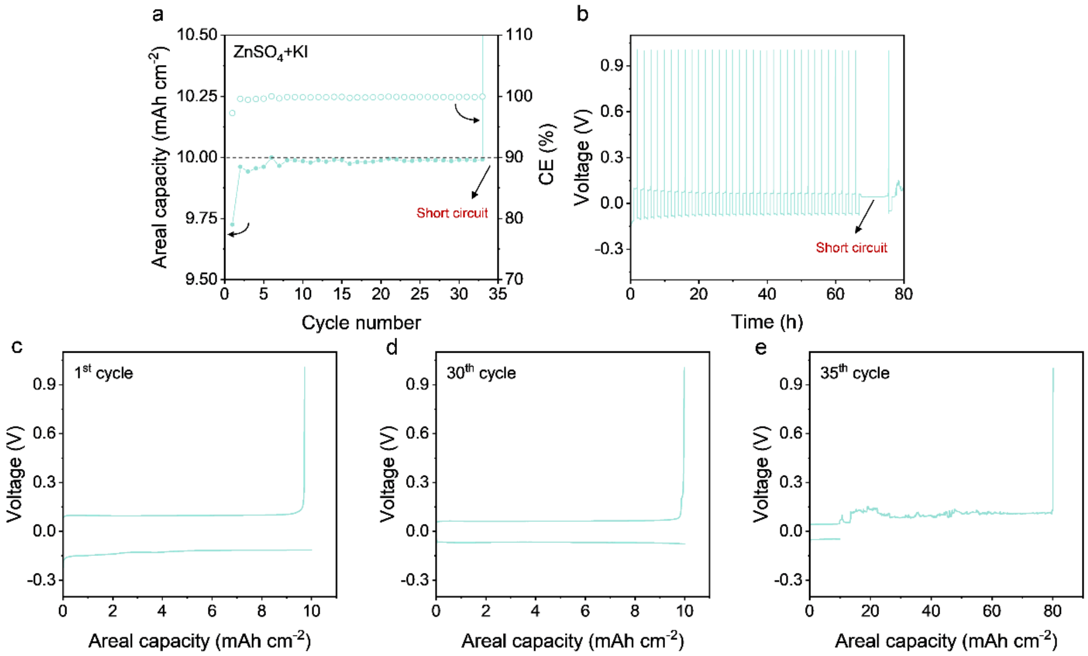


**Supplementary Fig. 35. a** Capacity-CE curves of a Zn||Cu asymmetric cell using $ZnSO_4$+KI electrolytes at 10 mA $cm^{-2}$ and 10 mAh $cm^{-2}$, tested at 30°C. **b** Corresponding time-voltage curve. Potential profiles of the **c** 1st, **d** 30th, and **e** 35th cycles.

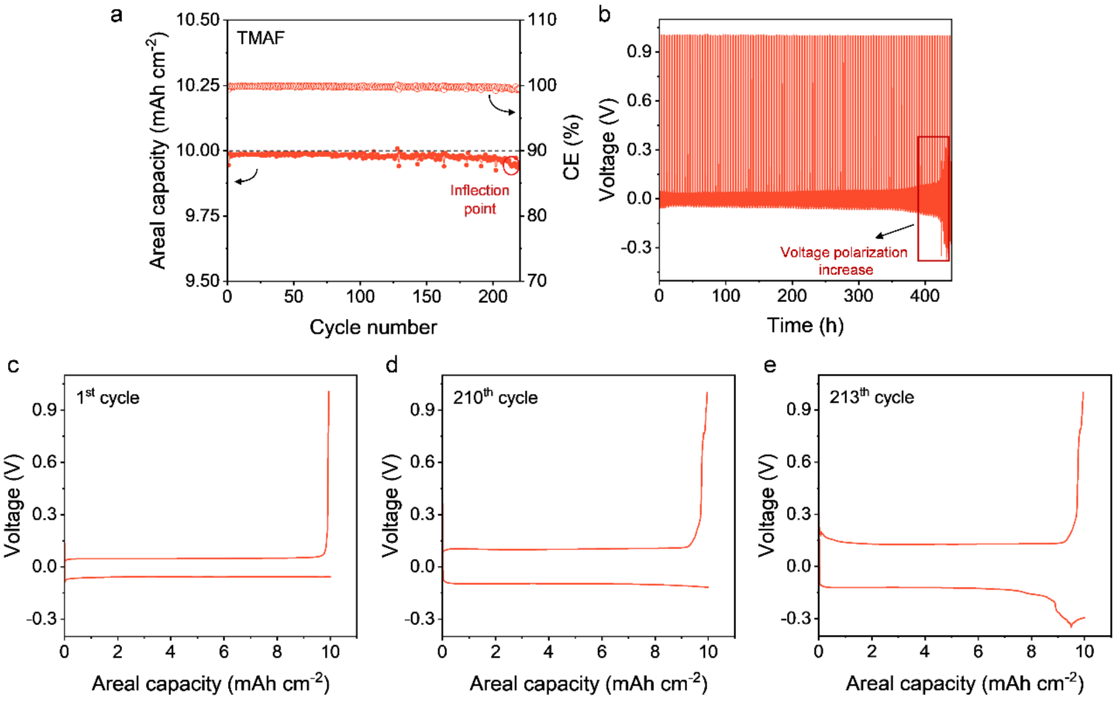


**Supplementary Fig. 36. a** Capacity-CE curves of a Zn||Cu asymmetric cell using TMAF electrolytes at 10 mA cm$^{-2}$ and 10 mAh cm$^{-2}$, tested at 30°C. **b** Corresponding time-potential curve. Potential profiles of the **c** 1$^{st}$, **d** 210$^{th}$, and **e** 213$^{th}$ cycles.

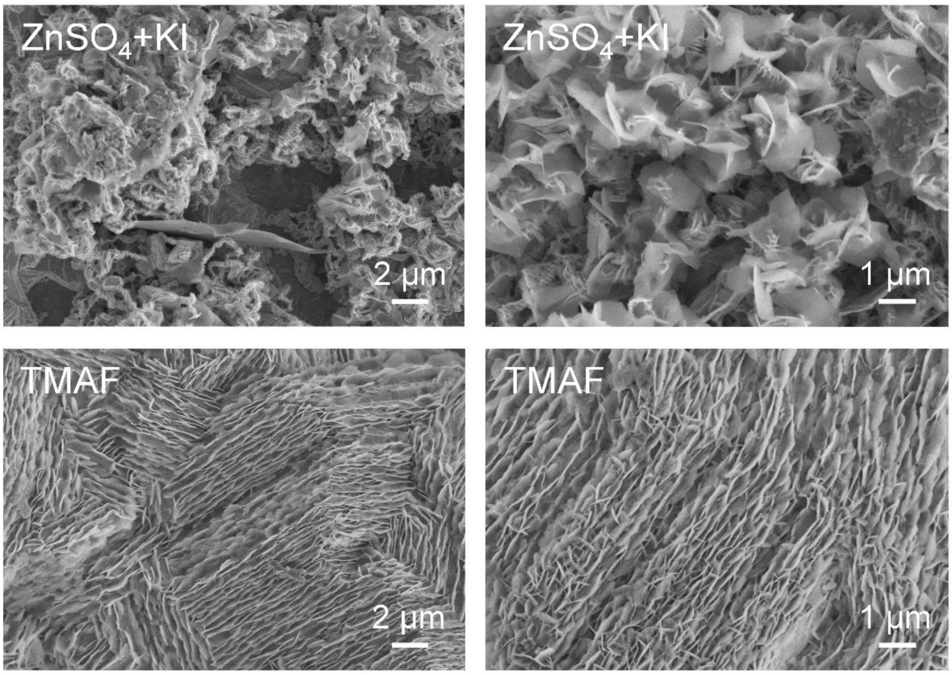


**Supplementary Fig. 37.** Surface morphology of Zn after 15 minutes of Zn deposition at 4 mA cm$^{-2}$ in $ZnSO_4$+KI and TMAF electrolytes.

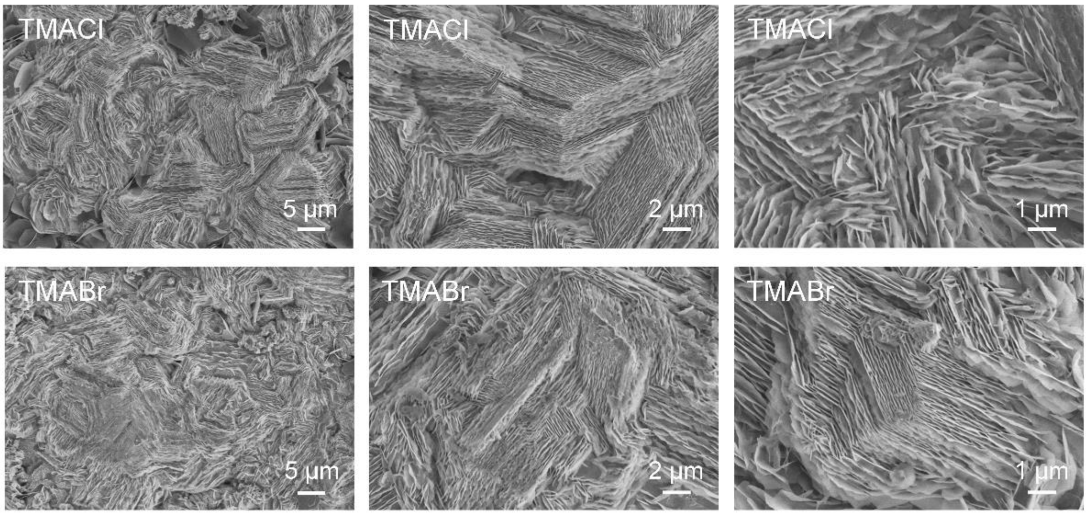


**Supplementary Fig. 38.** Surface morphology of Zn after 15 minutes of Zn deposition at 4 mA cm$^{-2}$ in TMACl and TMABr electrolytes.

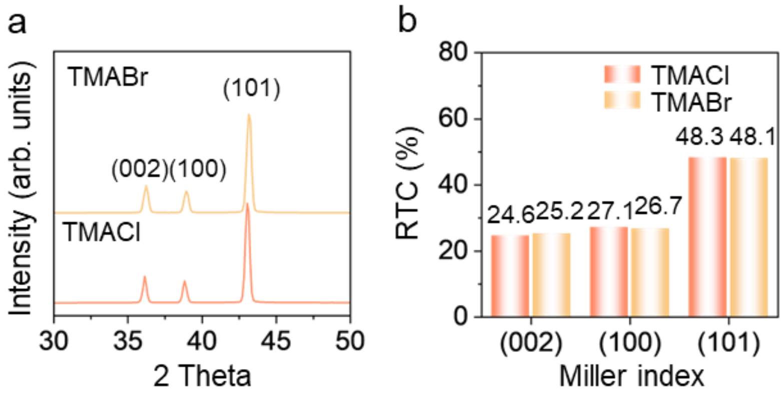


**Supplementary Fig. 39. a** XRD patterns and **b** the corresponding RTC and of Zn after 15 minutes of Zn deposition at 4 mA cm$^{-2}$ in TMACl and TMABr electrolytes.

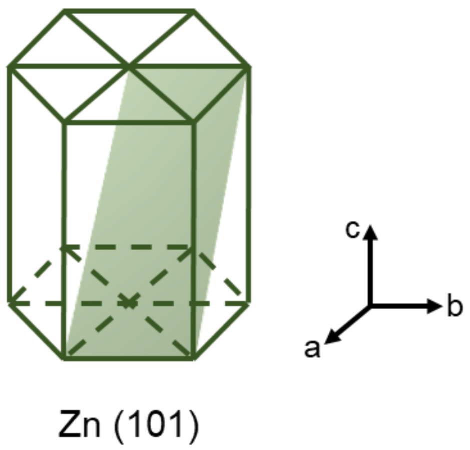


**Supplementary Fig. 40.** Schematic of Zn (101) plane.

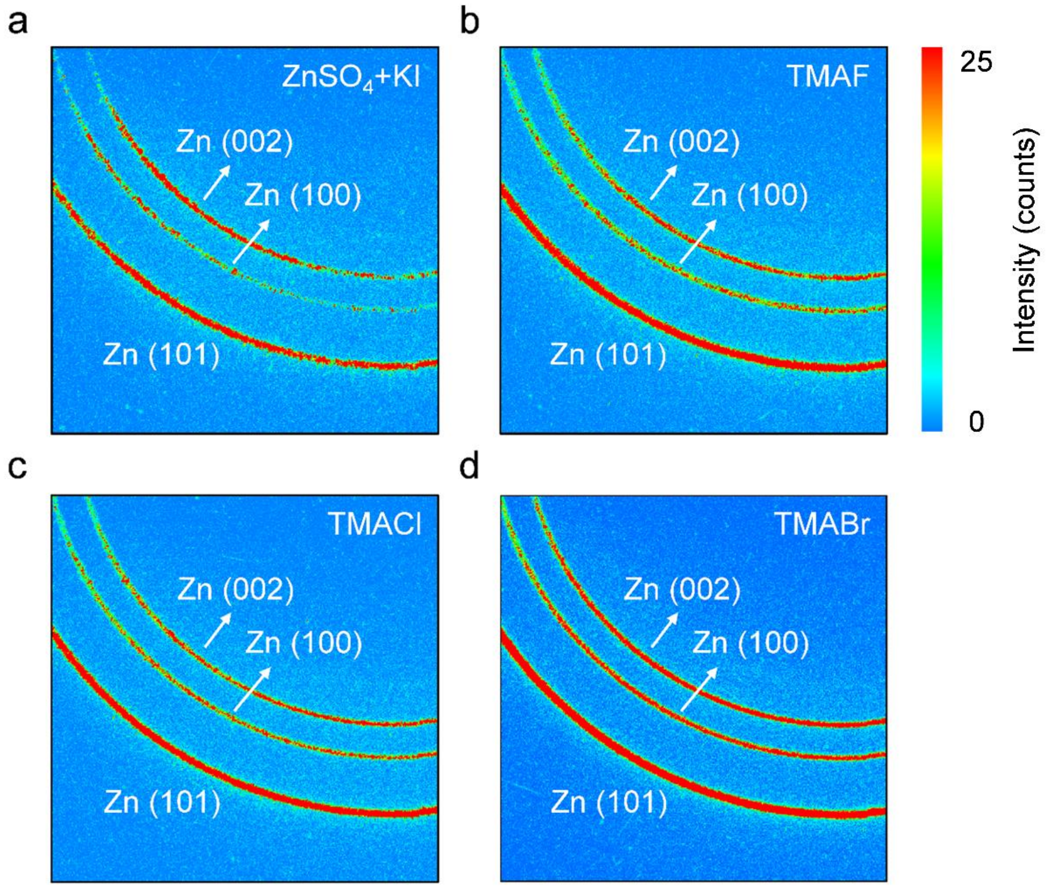


**Supplementary Fig. 41.** 2D GIWAXS patterns of deposited Zn negative electrodes after 15 minutes of Zn deposition at 4 mA $cm^{-2}$ using **a** $ZnSO_4$+KI, **b** TMAF, **c** TMACl and **d** TMABr electrolytes.

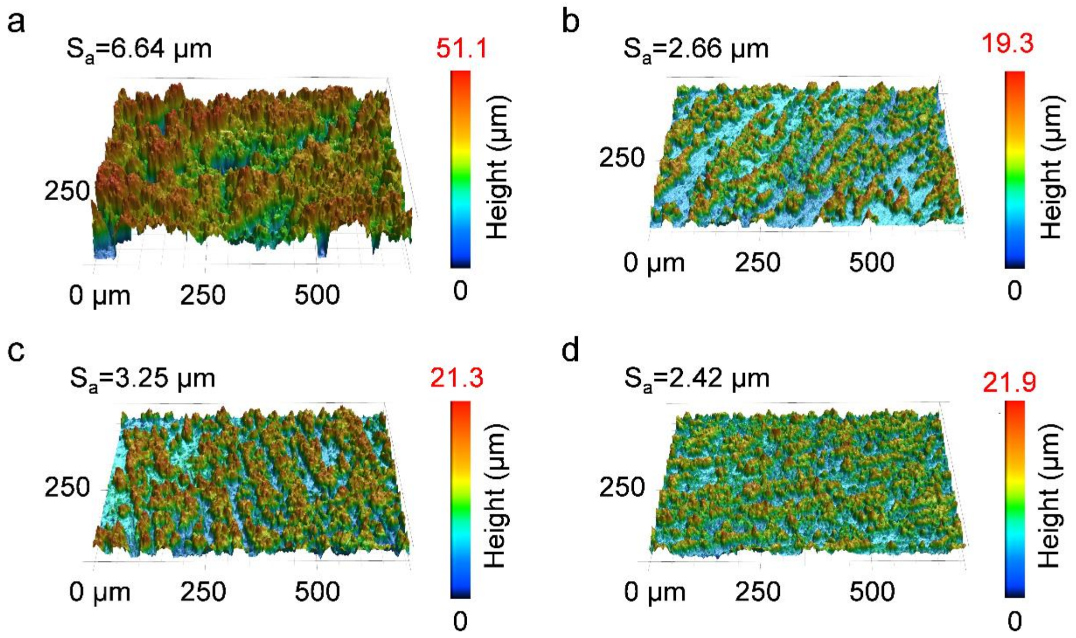


**Supplementary Fig. 42.** 3D confocal images and surface roughness ($S_a$) of deposited Zn after 1 hour's deposition at 4 mA cm$^{-2}$ in **a** $ZnSO_4$+KI, **b** TMAF, **c** TMACl and **d** TMABr electrolytes.

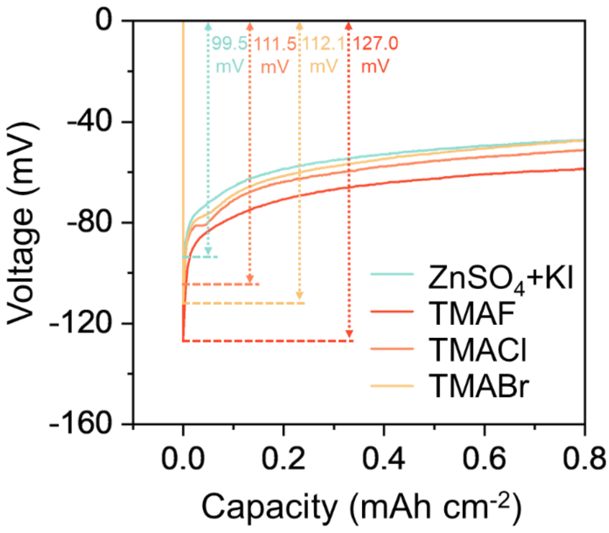


**Supplementary Fig. 43.** Nucleation overpotentials for cells using $ZnSO_4$+KI, TMAF, TMACl and TMABr electrolytes.

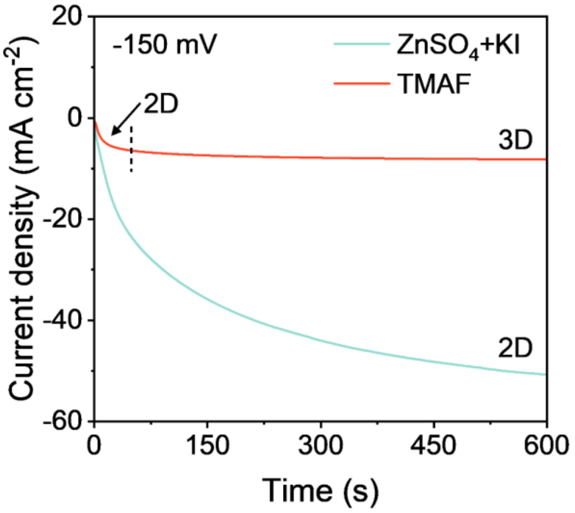


**Supplementary Fig. 44.** Chronoamperometric curves of Zn electrodeposition in different electrolytes under –150 mV.

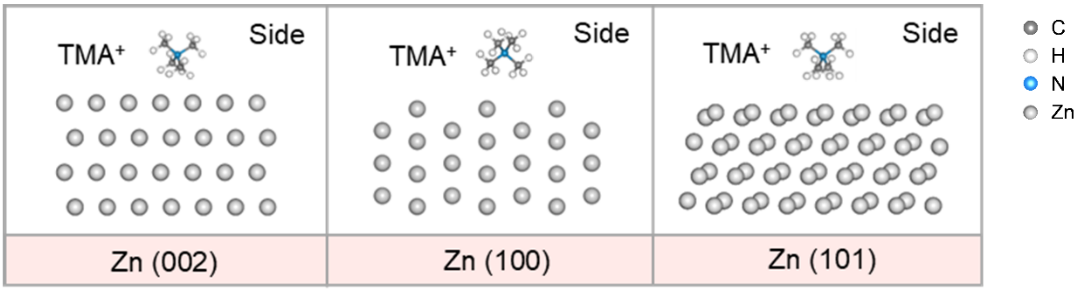


**Supplementary Fig. 45.** Adsorption of $TMA^+$ on different Zn planes.

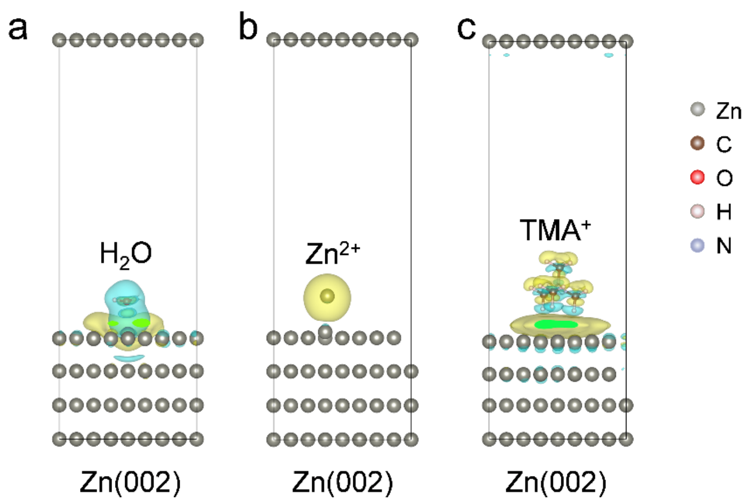


**Supplementary Fig. 46.** Differential charge density diagram of **a** $H_2O$, **b** $Zn^{2+}$ and **c** $TMA^+$ on Zn (002) planes. (Yellow indicates a rise in charge density, while blue indicates a fall in charge density.)

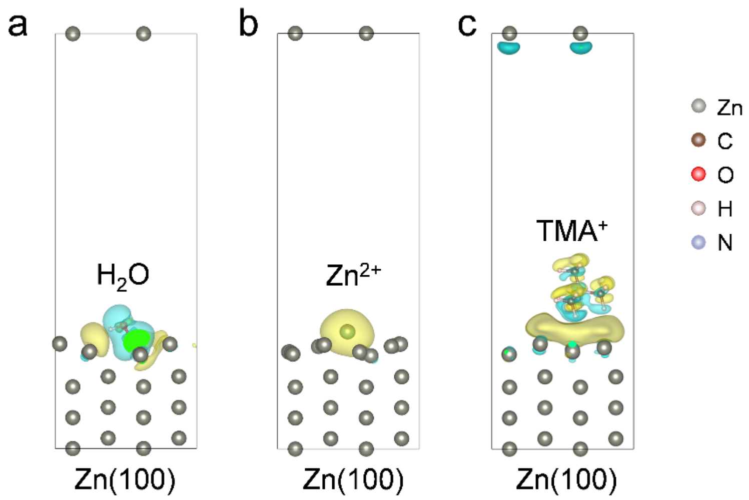


**Supplementary Fig. 47.** Differential charge density diagram of **a** $H_2O$, **b** $Zn^{2+}$ and **c** $TMA^+$ on Zn (100) planes.

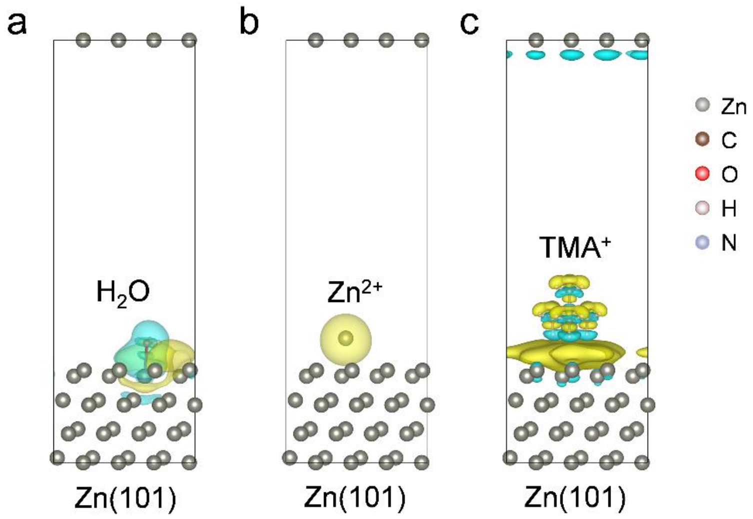


**Supplementary Fig. 48.** Differential charge density diagram of **a** $H_2O$, **b** $Zn^{2+}$ and **c** $TMA^+$ on Zn (101) planes.

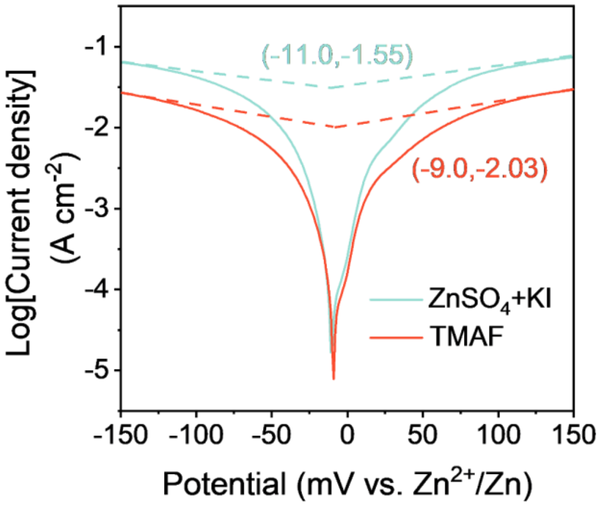


**Supplementary Fig. 49.** Tafel plots of Zn electrodes tested in electrolytes (scan rate=1 mV $s^{-1}$).

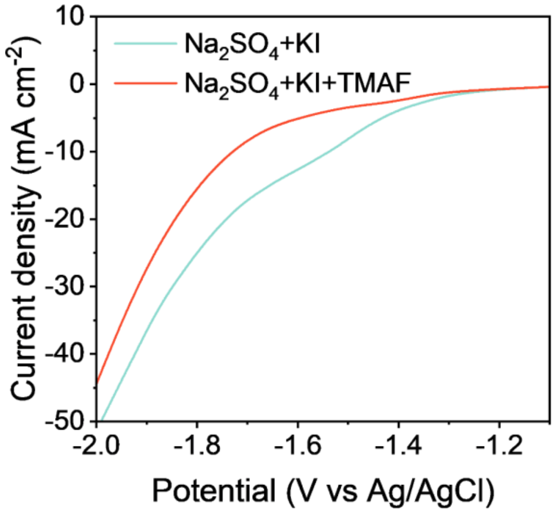


**Supplementary Fig. 50.** LSV characterization in the electrolytes (scan rate=5 mV $s^{-1}$).

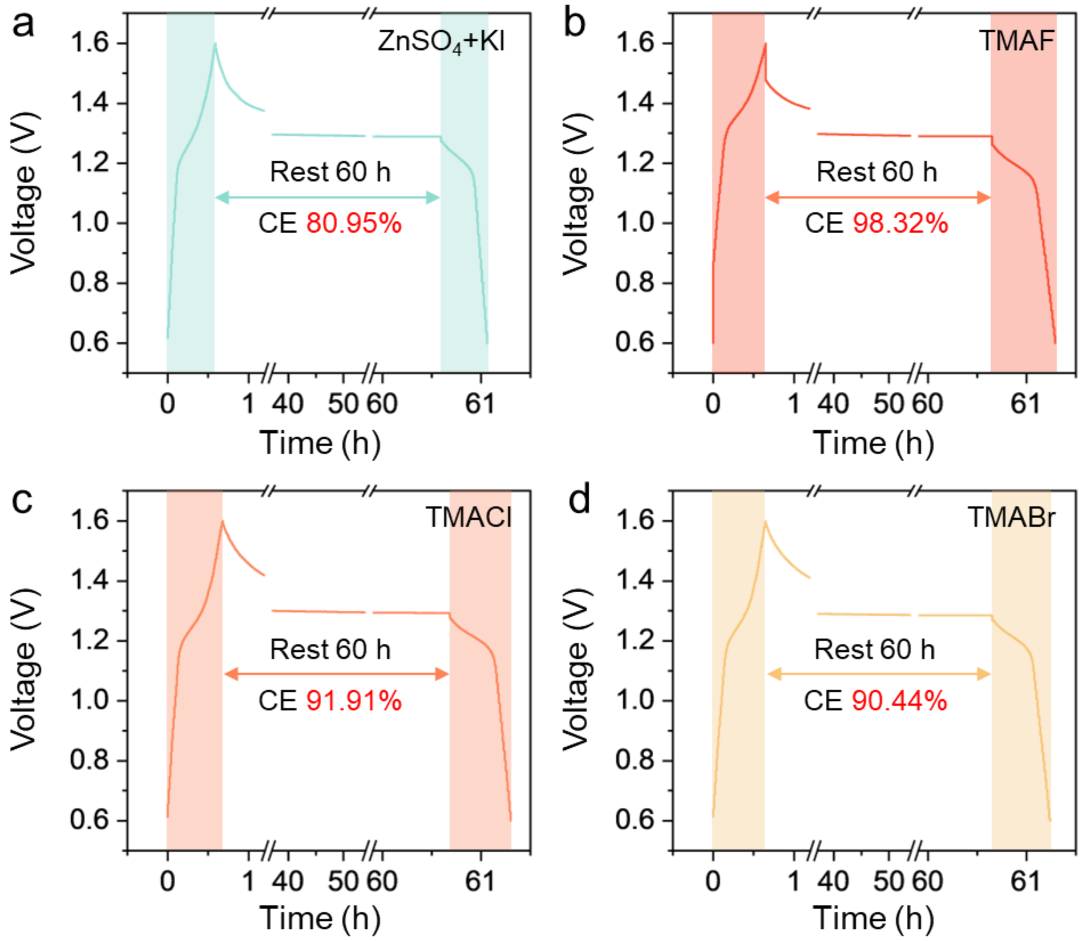


**Supplementary Fig. 51.** Self-discharge analysis using **a** $ZnSO_4$+KI, **b** TMAF, **c** TMACl and **d** TMABr electrolytes, tested at 30°C.

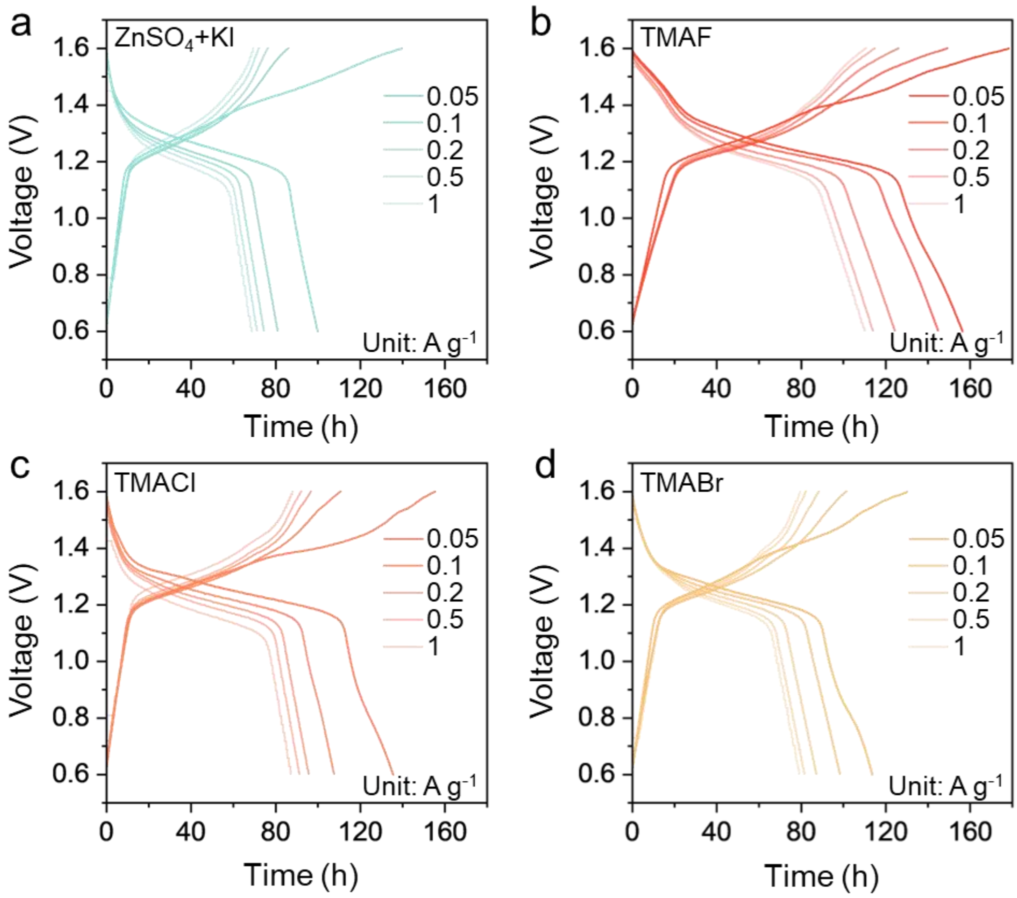


**Supplementary Fig. 52.** Charge/discharge curve for ZICBs using **a** $ZnSO_4$+KI, **b** TMAF, **c** TMACl and **d** TMABr electrolytes at different rates, tested at 30°C.

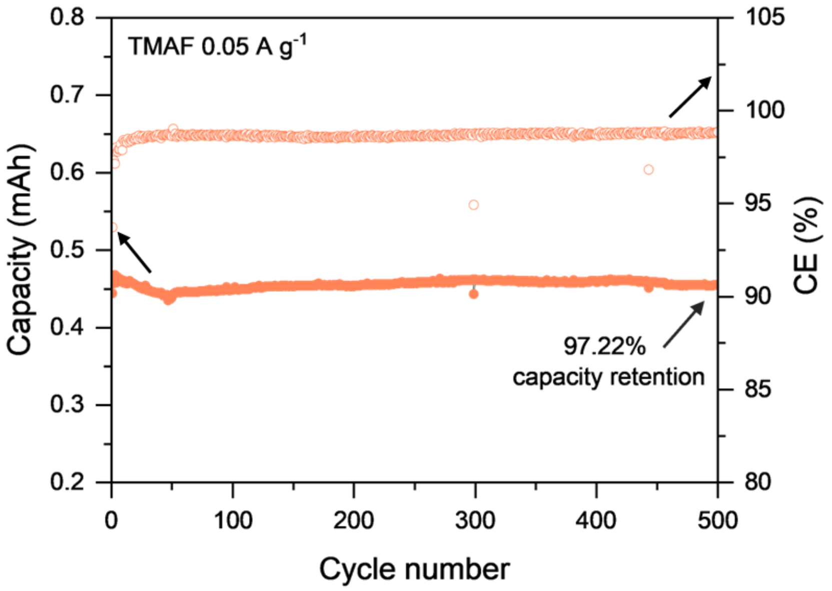


**Supplementary Fig. 53.** Cycling stability and CE profiles of ZICBs using TMAF electrolyte at a specific current of 0.05 A $g^{-1}$, tested at 30°C.

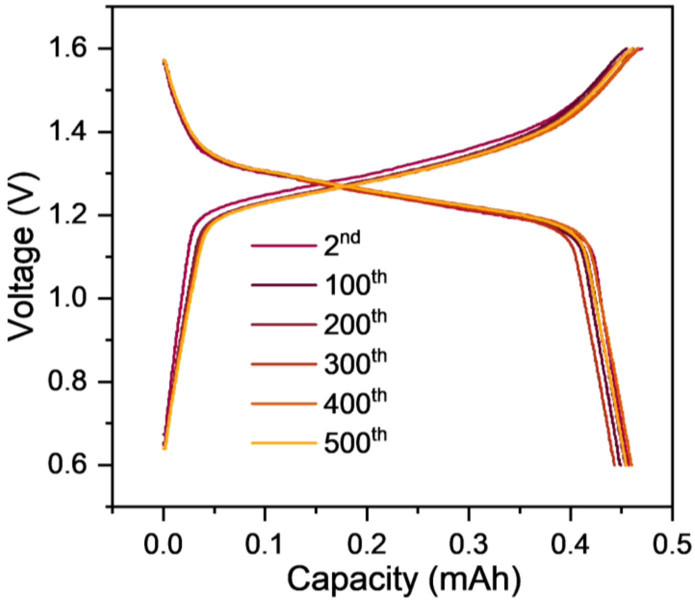


**Supplementary Fig. 54.** Charge/discharge curve for ZICBs using TMAF electrolyte at a specific current of 0.05 A $g^{-1}$, tested at 30°C.

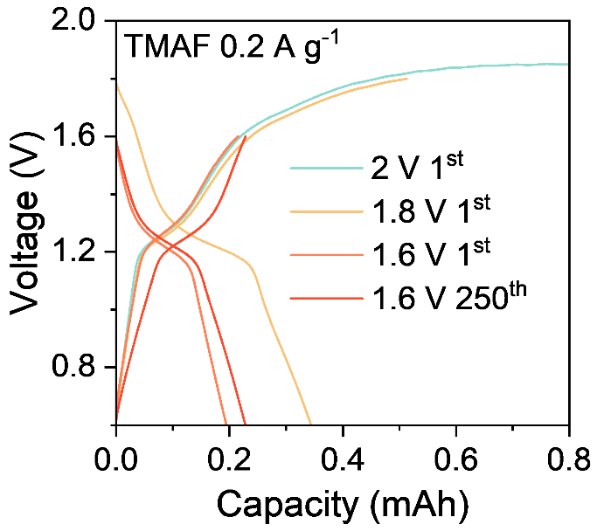


**Supplementary Fig. 55.** Charge/discharge curve for ZICBs using TMAF electrolytes at different potentials, tested at 30°C.

**Supplementary Note 1.** Calculation of apparent diffusion coefficient.

Apparent diffusion coefficient was calculated from CV measurements based on the equation 1 as follows:

$$I_p=2.69*10^5\, n^{3/2} A D_0^{1/2} v^{1/2}\, C \quad (1)$$

Here, $I_p$ represents the peak current, $n$ denotes the number of electrons transferred, $A$ is the area of the working electrode, $D_0$ stands for the diffusion coefficient of the electrolyte, $v$ represents the potential scan rate, and $C$ is the total concentration of the electrolyte.

**Supplementary Note 2.** Calculation of $I^-$ diffusion coefficient.

The diffusion coefficient of $I^-$ was calculated from GITT measurements based on the equation 2 as follows:

$$D=\frac{4L^2}{\pi\tau}\left(\frac{\Delta E_s}{\Delta E_t}\right)^2 \quad (2)$$

Here, $t$ refers to the duration of the applied current pulse (s), and $\tau$ represents the rest time (s). $\Delta E_s$ represents the steady-state potential change (V) by the current pulse. $\Delta E_t$ is the potential change (V) during the constant current pulse after eliminating the iR drop. $L$ is the ion diffusion distance (cm), which is equal to the electrode thickness.

**Supplementary Note 3.** Calculation of the relative texture coefficient (RTC).

RTC of each facet is calculated based on the equation 3 as follows:

$$RTC_{(hkl)}=\frac{I_{(hkl)}/I_{0(hkl)}}{\sum I_{(hkl)}/I_{0(hkl)}}\times 100\% \quad (3)$$

Here, $I_{(hkl)}$ means the intensity obtained from the actual sample, and $I_{0(hkl)}$ refers to the intensity of the standard-oriented sample. The relative intensities of $I_{0(hkl)}$ are from PDF#04-0831.

**Supplementary Table 1.** $S_{BET}$ and pore volume of activated carbon before and after charging in different electrolytes.

| Type of activated carbon | $S_{BET}$ ($m^2$ $g^{-1}$) | Pore volume ($cm^3$ $g^{-1}$) |
|---|---|---|
| Commercial activated carbon powder | 2152 | 0.94 |
| Uncharged | 509 | 0.27 |
| Charged to 1.6 V in $ZnSO_4$+KI electrolyte | 400 | 0.21 |
| Charged to 1.6 V in TMAF electrolyte | 379 | 0.20 |

**Supplementary Table 2.** Apparent diffusion coefficient measured by CV.

| Electrolyte | Process | Slope (k) | $D_0$ ($cm^2$ $s^{-1}$) |
|---|---|---|---|
| TMAF | Oxidation | 0.173 | $2.59\times10^{-6}$ |
| | Reduction | 0.190 | $3.12\times10^{-6}$ |
| TMACl | Oxidation | 0.159 | $2.18\times10^{-6}$ |
| | Reduction | 0.178 | $2.74\times10^{-6}$ |
| TMABr | Oxidation | 0.150 | $1.94\times10^{-6}$ |
| | Reduction | 0.173 | $2.59\times10^{-6}$ |

**Supplementary Table 3.** Gibbs free energies of $I_2X^-$ and $TMAI_2X$ and Gibbs free energy differences (ΔG) of step I and step II. (Unit: eV)

| X | $I_2X^-$ | $TMAI_2X$ | Step I (ΔG) | Step II (ΔG) |
|---|---|---|---|---|
| F | –4.11 | –6.57 | –4.11 | –2.46 |
| Cl | –3.37 | –4.74 | –3.37 | –1.37 |
| Br | –3.12 | –4.27 | –3.12 | –1.15 |
| I | –2.89 | –3.84 | –2.89 | –0.95 |

**Supplementary Table 4.** ACE, AVE and AEE in different electrolytes at a specific current of 0.2 A $g^{-1}$.

| Electrolytes | ACE (%) | AVE (%) | AEE (%) |
|---|---|---|---|
| $ZnSO_4$+KI | 95.3 | 92.0 | 87.8 |
| TMAF | **99.8** | **95.4** | **95.2** |
| TMACl | 99.6 | 95.3 | 94.9 |
| TMABr | 99.4 | 95.0 | 94.5 |

**Supplementary Table 5.** Energy efficiency of Zn-halogen batteries reported recently.

| Category | Strategy | Specific current (A $g^{-1}$) | Energy efficiency (%) | Reference |
|---|---|---|---|---|
| Zn-interhalide complexes batteries | Cation-driven phase transition and anion-accelerated kinetics strategy (TMAX electrolyte) | 0.20 | **95.2** | **This work** |
| | | 1.00 | **94.8** | |
| Static Zn–iodine batteries | Eutectic electrolyte | 0.16 | 92.9 | Ref. 22 |
| Static Zn–iodine batteries | Co@AC nanoreactor | 1.00 | 89.2 | Ref. 54 |
| Static Zn–iodine batteries | Nitrogen doped porous carbons | 1.00 | 89.0 | Ref. 55 |
| Zn–iodine flow batteries | Starch additive | 0.03 | 86.0 | Ref. 56 |
| | | 0.06 | 83.0 | |
| | | 0.09 | 78.0 | |
| | | 0.12 | 74.0 | |
| | | 0.15 | 70.0 | |
| Zn–iodine flow batteries | Ammonium-based electrolyte | 0.01 | 78.0 | Ref. 57 |
| Zn–iodine flow batteries | Porous polyolefin membrane | 0.35 | 81.0 | Ref. 58 |
| Zn–iodine flow batteries | Porous polyolefin membrane | 0.30 | 82.0 | Ref. 59 |
| Static Zn–bromine batteries | Polymer positive electrode | 1.14 | 74.0 | Ref. 60 |
| Zn-bromine flow batteries | Carbon felt-based electrode with N-rich defects | 0.03 | 82.0 | Ref. 61 |
| | | 0.07 | 65.4 | |
| | | 0.08 | 63.1 | |
| Zn-bromine flow batteries | Cage-like porous carbon | 0.20 | 81.0 | Ref. 62 |

**Supplementary Table 6.** CE, VE and EE for ZICBs using TMAF electrolytes at different potentials at 0.2 A $g^{-1}$.

| | Potential (V) | Efficiency (%) |
|---|---|---|
| CE | 1.6 (250th cycle) | **99.9** |
| | 1.6 (1st cycle) | 90.4 |
| | 1.8 (1st cycle) | 67.1 |
| | 2.0 (1st cycle) | - (unable charge to 2 V) |
| VE | 1.6 (250th cycle) | **95.6** |
| | 1.6 (1st cycle) | 88.7 |
| | 1.8 (1st cycle) | 78.8 |
| | 2.0 (1st cycle) | - (unable charge to 2 V) |
| EE | 1.6 (250th cycle) | **95.5** |
| | 1.6 (1st cycle) | 80.2 |
| | 1.8 (1st cycle) | 52.9 |
| | 2.0 (1st cycle) | - (unable charge to 2 V) |
