# Supplementary material for: Cation-driven phase transition and anion-enhanced kinetics for high energy efficiency zinc-interhalide complex batteries
Source: Nat Commun. 2025 May 17;16:4586. doi: 10.1038/s41467-025-59894-w (PMC12085677; doi:10.1038/s41467-025-59894-w)
Supplement: Supplementary file 3 — Description of Additional Supplementary Files [file 41467_2025_59894_MOESM3_ESM.pdf]

Description of Additional Supplementary Files

Supplementary Data 1: The Gibbs free energy calculation model of I_2X^- and $TMAI_2X$.

Supplementary Data 2: Valence states (atomic Mullikan charge) calculation model of I_2X^-.

Supplementary Data 3: The adsorption model of the carbon layer for $TMAI_2X$.
